# Awareness and Knowledge About Preconception Healthcare: A Cross-Sectional Study of Early Years UAE Medical Students

**DOI:** 10.3390/jcm14010181

**Published:** 2024-12-31

**Authors:** Sara Maki, Shamsa Al Awar, Sara Alhosani, Latifa Alshamsi, Shamma Alzaabi, Mohammad Ali Alsaadi, Mahra Alhammadi, Hamad Alhosani, Gehan Sayed Salam, Stanisław Wójtowicz, Kornelia Zaręba

**Affiliations:** 1 Department of Obstetrics & Gynecology, College of Medicine & Health Sciences (CMHS), United Arab Emirates University, Al Ain P.O. Box 15551, United Arab Emirates; sara.maki@uaeu.ac.ae (S.M.); sawar@uaeu.ac.ae (S.A.A.); gsayed@uaeu.ac.ae (G.S.S.); 2 College of Medicine & Health Sciences (CMHS), United Arab Emirates University, Al Ain P.O. Box 15551, United Arab Emirates; sara.alhosani147@gmail.com (S.A.); latifa.y.alshamsi@hotmail.com (L.A.); 201900424@uaeu.ac.ae (S.A.); mohammadalialsaadi01@gmail.com (M.A.A.); mahraalhammadi93@gmail.com (M.A.); hamad.alhosani100@gmail.com (H.A.); 3 Department of Health Psychology, Medical University of Warsaw, 02-091 Warsaw, Poland; stwojt@o2.pl; 4 Institute of Psychology, Old Polish University of Applied Sciences, 25-666 Kielce, Poland

**Keywords:** medical students, preconception, knowledge, preconceptional health, pregnancy preparation, medical knowledge, student’s knowledge, preconceptional awareness

## Abstract

**Background:** Preconception health is critical for improving maternal and child health. The main objective of the study was to explore medical students’ health habits, quality of life, and knowledge of preconception healthcare. **Methods:** We conducted a cross-sectional study between 15 March 2023 and 31 May 2024 among medical students at United Arab Emirates University. To determine awareness and knowledge of preconception health, we administered a survey consisting of an author’s questionnaire with 35 questions covering sociodemographic characteristics and general knowledge of preconception health, as well as the WHO Quality of Life Scale-BREF (WHOQOL-BREF). **Results:** The participants were predominantly under 25 years old (98.5%), Emirati (91.1%), single (92.6%), and female (95.8%); only 3.4% had been pregnant before. Regarding health awareness and behaviors, a significant number of females (58.0%) had never visited a gynecologist. The majority of students (72.4%) participated in sports activities. The overall level of knowledge was low, with a mean level of 7.5 (SD = 6.36) out of 24. The Internet (webpages, blogs, webinars) (64.5%) was the major source of knowledge regarding healthcare information, followed by social media platforms (Twitter, Facebook, TikTok, Instagram) and mobile applications (57.5%), books (48.6%), and family members (57.0%). There was a statistically significant correlation between knowledge levels and the Internet (*p* < 0.004) or family (*p* < 0.001) as a source of knowledge. Additionally, there was a statistically significant positive correlation between knowledge and quality of life across all four WHOQOL domains. **Conclusions:** Medical knowledge might positively affect general well-being. Fostering stronger social networks and support systems could benefit preconceptional awareness and knowledge.

## 1. Introduction

Improving maternal health and reducing child mortality are global health objectives that support the Millennium Development Goals [1]. Active preparation for pregnancy is associated with healthy lifestyles in both women and men during the preconception period [2]. Multiple risk factors, such as smoking and drinking, as well as diseases such as anemia or psychiatric diseases, can be diagnosed and managed before pregnancy to achieve optimal well-being for the future mother [3]. Against this background, preconception health emerged as a field of medicine [4].

Preconception care involves interventions made before pregnancy intended to enhance the health of women and couples while improving pregnancy and child health results [5,6]. It is an umbrella term that refers to health promotion, risk assessment, and initiating interventions to target risk factors that potentially influence pregnancy outcomes. The goal of preconception health research is to use preventive behavior and healthcare to optimize the health of future offspring arising from both planned and unplanned pregnancies [7,8]. Therefore, awareness of potential threats is crucial for society as a whole.

The general well-being of both partners before marriage is the goal of preconception health. Programs for preconception health can include informing partners about chronic medical conditions, such as diabetes, hypertension, and STDs, which can have adverse effects on pregnancy [9]. It is also crucial to maintain a healthy lifestyle, which includes exercise, a balanced diet, and a healthy weight. Moreover, vaccinations help protect fetuses from disease [10]. Given the high percentage of consanguineous marriages in the United Arab Emirates (UAE), a vital component of preconception health is genetic counseling and the diagnosis of diseases such as thalassemia and sickle cell diseases.

Medical students would seem to be the best group of medical practitioners for introducing appropriate habits to patients from the early stages of their professional careers. Therefore, sufficient knowledge of preconception care is vitally important among medical students [11]. In the UAE, medical students gain knowledge about preconception care during their clinical years, usually during OBGYN or public health clinical rotations. In the United Arab Emirates University (UAEU) College of Medicine and Health Sciences, it is only taught thoroughly during the OBGYN clinical clerkship, which occurs around the fifth year of students’ six-year MD program. It is vital, then, to enhance preconception health knowledge among medical students, doctors, and the community.

Delays in introducing this important healthcare service are expected to affect knowledge and attitudes toward preconception care among medical students in the UAE. Hence, the present study aimed to provide a means to evaluate the amount or degree of knowledge that UAEU medical students possess regarding preconception care to identify knowledge gaps and develop targeted educational interventions to improve preconception care.

The main objectives of the study were:

1. Explore medical students’ knowledge of preconception healthcare, pregnancy, and health behaviors among early years medical students;
2. Explore sources of knowledge regarding preconception healthcare;
3. Provide an overview of the barriers that affect the provision of preconception healthcare.

## 2. Materials and Methods

### 2.1. Study Design and Setting

This cross-sectional study was designed to determine awareness and knowledge of preconception health among UAEU medical students. It was conducted between 15 March 2023 and 31 May 2024.

### 2.2. Study Population

The studied population were young adult male and female medical students who consented to participate in the study.

Inclusion Criteria:

1. Medical student at UAEU (years 1–4);
2. Of reproductive age (18–45 years of age).

Exclusion Criteria:

1. Lack of consent to participate in the study;
2. Lack of understanding of English or Arabic.

### 2.3. Data Collection

We selected qualitative methods because they are more conducive to exploring students’ experiences and understanding their health and information needs. We administered a survey consisting of an author’s questionnaire with 35 questions covering sociodemographics and general knowledge about preconception health, as well as the WHO Quality of Life Scale-BREF (WHOQOL-BREF).

We sent an online questionnaire created in Google Forms to all targeted UAEU medical students (*n* = 625). The questionnaire was written in English and then translated into Arabic. The first section of the questionnaire was the consent section, in which participants agreed to participate in the study. The response rate was 34.24%.

**Figure 1:**
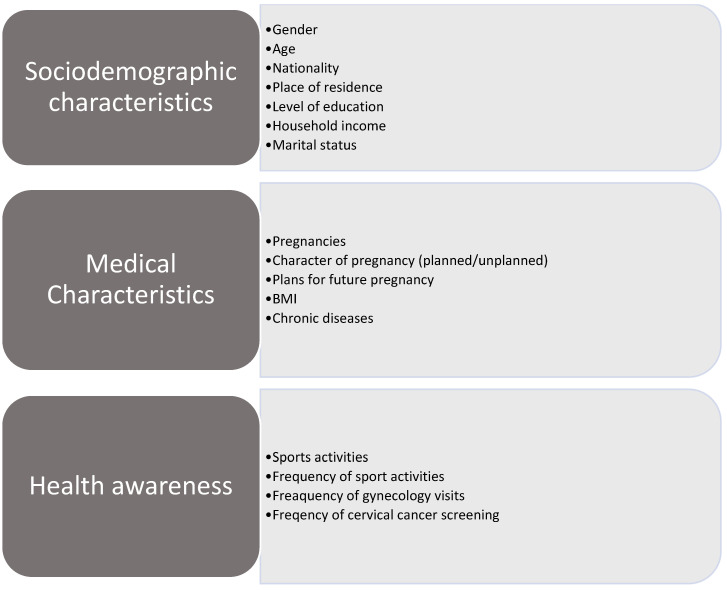
Questionnaire description- sociodemographic and medical characteristics.

The data obtained from the questionnaire consisted of sociodemographic parameters and medical data, including pregnancy-related and lifestyle information (Figure 1).

Moreover, we described the level of knowledge that was calculated using the following Likert scale:

- Strongly agree/disagree depending on whether the statement was correct: +2 or −2 marks (+2 for correct answers and −2 for incorrect answers);
- Agree/disagree depending on whether the statement was correct (+1 for correct answers, −1 for incorrect answers);
- I don’t know: 0 marks.

In total, we obtained scores ranging from −24 to 24. We established the following cutoff points for the level of knowledge:

- 12–24 good level of knowledge (3);
- 0–12 low level of knowledge (2);
- 0 to −24 no knowledge (1).

**Figure 2:**
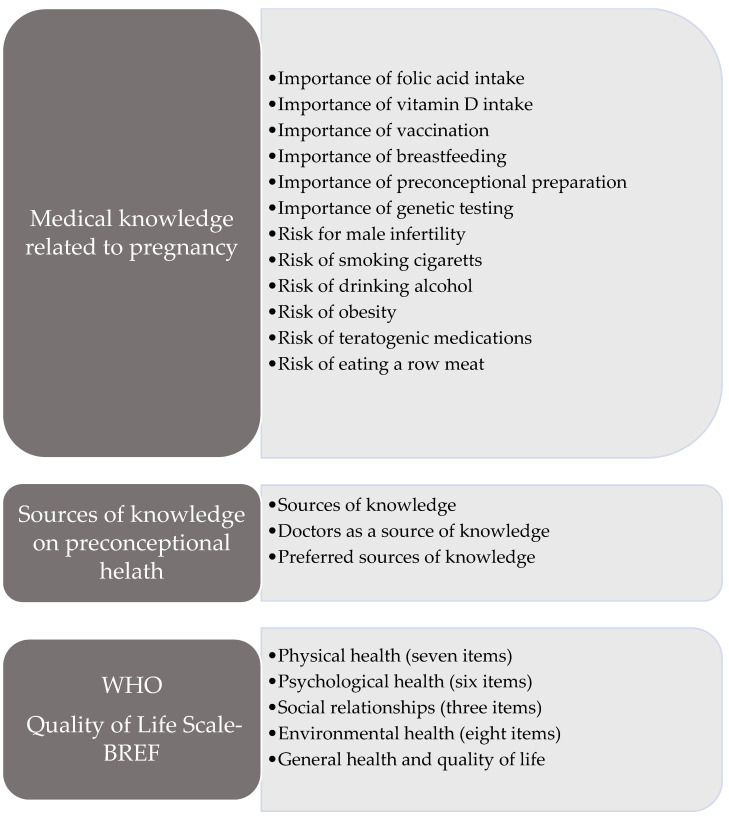
Questionnaire—knowledge and quality of life.

Finally, we documented knowledge sources, including where participants found healthcare information and their preferred sources of knowledge. Baseline measurements were obtained after the questionnaire was completed (Figure 2).

To maintain data collection quality, we closely monitored the process and provided support or clarification to participants if they encountered any difficulties or had questions. We established procedures to ensure the secure storage of the completed questionnaires, maintain confidentiality, and protect the privacy of the participants, ensuring that no personal data were collected.

#### WHO Quality of Life Scales

WHOQOL was developed by the WHOQOL Group, along with 15 international field centers, to establish a quality-of-life (QoL) assessment tool that would be cross-culturally applicable [12]. It assesses overall QoL by examining four critical domains: physical health, psychological health, social relationships, and environmental factors (Figure 2). The WHOQOL-BREF is a 26-item instrument consisting of four domains: physical health (seven items), psychological health (six items), social relationships (three items), environmental health (eight items), QoL, and general health. Each item of the WHOQOL is scored on a five-point ordinal scale. The scores are then linearly transformed into a scale of 0–100 [12]. The scale has very good psychometric properties [12]. A validation of 3400 multinational respondents living in Singapore revealed good internal consistency, as evidenced by high alpha coefficients for the physical (0.79), psychological (0.82), social relationship (0.81), and environmental (0.83) domains [13]. In the present study, data from 214 participants were analyzed to obtain insights into how these areas affected their well-being.

### 2.4. Statistical Analysis

The independent variables in the survey included demographic, medical, pregnancy-related, and lifestyle factors; the dependent variables included participants’ medical knowledge and opinions regarding various aspects of preconception and pregnancy care. Age, height, and weight were numerical variables, whereas body mass index (BMI) and sex were categorical variables.

Categorical data such as sex and BMI are presented as frequencies. We calculated prevalence using data from the questionnaire. A chi-square test was used to assess the association between medical students’ preconception knowledge and other categorical risk factors. Kendall’s tau correlation coefficient was used to associate WHOQOL and BMI (co-occurrence). We examined the relationship between WHOQOL and knowledge levels using Pearson’s r correlation.

The statistical software SPSS version 28.0 was used for data entry and analysis (IBM SPSS Statistics, 2021, Armonk, NY, USA). Statistical significance was set at *p* < 0.05.

### 2.5. Ethical Aspects

This study was conducted in accordance with the Declaration of Helsinki and approved by the Institutional Bioethics Committee of the UAEU College of Medical Health Sciences (approval no. ERSC_2022_2233).

## 3. Results

### 3.1. Sociodemographic Characteristics

The study population comprised 214 individuals, predominantly women (95.8%), with a small proportion of men (4.2%). The majority of the participants were under 25 years of age, with 50.5% under 20 years and 48.0% between 20 and 25. The sample was predominantly Emirati (91.1%), reflecting a strong representation of the UAE. Notably, the majority resided in Abu Dhabi (74.3%), followed by Fujairah (5.6%) and Ras Al Khaimah (7.0%) (Table 1). Most participants had completed secondary school (32.7%) or held a bachelor’s degree (37.4%), with a smaller fraction having attained a diploma (26.6%). Household income varied, with a significant portion earning less than 50,000 AED annually (33.2%). The study population was predominantly single, comprising 91.6% of the participants, with a small percentage married (7.0%).

### 3.2. Medical Characteristics

Regarding medical characteristics, responses revealed that only seven female respondents (3.4%) had been pregnant before, with the majority having never been pregnant (91.7%) (Table 2). Only 1.9% had two children, and 0.5% had eight children, while the majority (27.6%) reported having no children; however, we had no data regarding children for 70.1% of the participants. These findings are justifiable because most participants were single (91.6%). Most participants (78%) had no inherited genetic conditions, and 22% declared they had genetic diseases in their families. Approximately 20 participants (9.3%) reported being ill (Table 2).

Regarding the planned character of the pregnancy, we received inconsistent data that suggests that the students did not understand the question properly (too many students claimed their pregnancy was planned or unplanned in comparison with the question on the number of pregnancies). Regarding future pregnancy plans, 28.5% of the participants had plans to get pregnant at present, and 23.4% did not plan to get pregnant at all. By contrast, 12.6% were considering pregnancy in the next 3–5 years and 11.2% in the next 1–2 years (Table 2).

To assess health awareness and the implementation of that knowledge, we asked the participants about sports activities and the frequency of regular medical checkups. Questions assessing participants’ physical activity awareness revealed that 72.4% participated in sports activities while 27.6% did not. Of these, 33.2% reported engaging in sports activities a few times a week, and 15.9% did so daily. Regarding women’s health awareness, a significant percentage (58.0%) of respondents reported that they had never visited a gynecologist, with only 26.8% visiting an OB-GYN at least once a year. As for Pap smears, a large majority of females (78%) had never had a Pap smear, with only 13.7% reporting receiving a Pap smear at least once every three years and 6.8% undergoing a Pap smear at least once a year. Moreover, we asked the participants about their weight and height and calculated their BMI. The median BMI was 22.92 (SD = 4.92) with a minimum of 13.96 and a maximum of 41.14.

### 3.3. Sources of Knowledge

**Figure 3:**
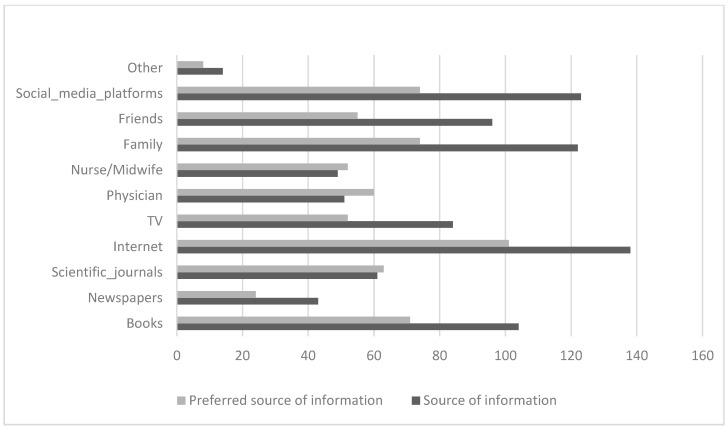
Sources of information and preferable sources of information (*n* = 214).

The analysis of participants’ preferences when selecting information sources showed a clear trend in which most relied on the Internet (webpages, blogs, webinars), with most participants (64.5%) using the Internet to access healthcare information (Figure 3). Social media platforms and mobile applications also played a crucial role, with 57.5% of respondents using Facebook, Twitter, or Instagram for health-related information. Other traditional sources, such as books (48.6%) and information from family members (57.0%), were almost equally important.

Regarding the sources that participants preferred to use to gain medical knowledge about preconception health, there was a clear preference for mobile applications (50.7%). Instagram, Twitter, and Facebook were a close second, accounting for 41.9% of the participants. Webpages and blogs were also popular, selected by 36.3% and 23.7% of the participants, respectively. Webinars were chosen by 16.7% of participants, whereas a smaller percentage (6.0%) opted for other sources. Regarding the number of sources used, many respondents preferred using one (16.4%) or two (11.7%) sources to access healthcare-related information. However, diversity was also common when accessing information, including three (13.1%), four (17.8%), and five (15.4%) sources.

Regarding the number of sources used for learning, most participants (52.8%) indicated a preference for using a single source. A significant proportion (24.3%) favored knowledge acquisition from two sources, whereas 18.2% chose to acquire knowledge from three sources. By contrast, a smaller proportion of participants used four (3.3%) or five (1.4%) sources for knowledge acquisition.

### 3.4. Levels of Knowledge

Awareness of lifestyle choices that affect fertility and pregnancy varies. Many participants recognized the adverse effects of smoking and alcohol consumption during pregnancy (Table 3). Additionally, there was a strong acknowledgment of the importance of vitamin D and the effects of obesity in the preconception phase. Many understood the need for medication adjustments during pregnancy and the risks associated with eating raw meat. Knowledge about the benefits of infant formula relative to breastfeeding was widespread, and many emphasized the importance of preparing for pregnancy, even among healthy individuals. However, there was a notable lack of understanding regarding the importance of vaccinations before pregnancy and the critical role of folic acid, with 42.5% and 45.3% of participants answering, “I don’t know”. Additionally, 39.3% of the participants did not know whether decreasing obesity was important during the preconception period. Moreover, approximately one-third of the participants had no awareness of the importance of changing unsafe medicaments before pregnancy (31.3), the effects of eating raw meat during pregnancy (36%), or the influence of an unhealthy lifestyle on fertility (32.7%) (Table 3).

The overall level of students’ knowledge was low, with only 29% demonstrating good understanding and 22% showing a lack of knowledge about preconception health (Table 4). The median level of knowledge was 7.5 (SD = 6.36) out of 24 with a minimum result of −9.

### 3.5. Quality of Life

The highest QoL score was observed in the environmental domain, with a median of 112.30 (SD = 27.18), or 70% of the maximum, with scores ranging from 32 to 160. The physical health domain had a median score of 93.40 (SD = 18.14), representing about 67% of the maximum possible score, with scores ranging from 48 to 140 (Table 5). The psychological health domain had a median score of 78.13 (SD = 16.67), or 65% of the maximum, with scores ranging from 40 to 120. The social relationships domain had a median score of 40.45 (SD = 10.33), corresponding to 67% of the maximum score, ranging from 12 to 60.

### 3.6. Correlation Between WHOQOL and BMI

The correlation between the levels of all domains of the WHOQOL and BMI, as measured by the Tau-b Kendall test, showed no statistical significance. The p-values for the physical domain, the psychological domain, social relationships, and the environmental domain were 0.318, 0.802, 0.595, and 0.698, respectively.

### 3.7. Correlation Between Levels of Knowledge and Sources of Information

The chi-square test indicated a statistically significant positive correlation between the level of knowledge when the sources were the Internet (*p* < 0.004) and family (*p* < 0.001). However, this correlation was not observed for other sources such as books (*p* = 0.445), newspapers (*p* = 0.106), scientific journals (*p* = 0.025), TV (*p* = 0.682), physicians (*p* = 0.550), nurses/midwives (*p* = 0.319), friends (*p* = 0.027), or social media (*p* = 0.008).

### 3.8. Correlation Between Levels of Knowledge and Quality of Life

The Pearson’s correlation coefficient regarding the level of knowledge and QoL showed a statistically significant positive influence of knowledge on QoL across all four domains, with a *p*-value of 0.01 for each domain (Table 6).

## 4. Discussion

Nascimento et al. emphasized the importance of preconception care education [11]. Improving future maternal and child health outcomes will depend highly on medical students’ knowledge and attitudes about preconception health. Although this topic is important, few studies have described the preconception knowledge levels of this group. Most studies contribute to our understanding of preconception health knowledge among the general population [14,15,16]. However, the target audiences and assessment methods of those studies led to differences in the findings. Comparing them can offer a broader perspective on knowledge gaps and areas needing improvement in preconception health education. The present study revealed medical students’ level of knowledge regarding preconception healthcare and pregnancy and explored their attitudes and health behaviors.

This study’s sample was mainly young (98.5% under 25 years of age), Emirati (91.1%), single (92.6%), and female (95.8%), with only 3.4% having been pregnant before. Regarding health awareness and behaviors, a significant number (58.0%) of female respondents had never visited a gynecologist, but 72.4% of participants had participated in sports activities. The Internet (64.5%) was their major source of knowledge regarding healthcare information, followed by social media platforms (57.5%), family members (57.0%), and books (48.6%). The preferred source of knowledge was the Internet (47%), mainly by way of mobile applications (50.7%). There was a notable lack of understanding regarding the importance of vaccinations before pregnancy and the critical role of folic acid in preconception care, even among medical students. Additionally, about one-third of the participants were unaware of the importance of decreasing obesity in the preconception period (39%), changing unsafe medications before pregnancy (31.3%), the effect of eating raw meat during pregnancy (36%), and the influence of an unhealthy lifestyle on fertility (32.7%) (Table 4). Moreover, the overall level of knowledge was low, with a median of 7.5 (SD = 6.36) out of 24. The overall QoL in the experimental group was high in all WHOQOL domains, with the highest score in the environmental domain. There was a statistically significant correlation between the level of knowledge and the Internet (*p* < 0.004) or family (*p* < 0.001) as a source of knowledge. Additionally, there was a statistically significant positive influence across all four WHOQOL domains.

Our respondents were predominantly young, single, Emirati, and female and had completed secondary school or higher (96.7%). The high number of females reflects the culture of the UAE, where males are more commonly sent to study abroad. Moreover, medical studies in many other countries also tend to be feminized [17]. Similarly, research by Al-Hossani et al. on pregnant women in the UAE presented a relatively young group of participants (64.6% were aged 20–29) [18]. Despite similar ages, we had more single women in our study, which could have been attributable to their chosen life patterns. Most students get married after completing their studies [19]. The high proportion of young and educated participants in our study likely contributed to their greater awareness of preconception health compared with the general population [14]. The predominance of female participants could have also influenced the results, as women are generally more engaged in reproductive health [20]. Additionally, socioeconomic diversity, with 33.2% reporting household incomes below 50,000 AED annually, suggests a broad representation across economic strata, potentially influencing access to and attitudes toward preconception care. The high proportion of single participants might have affected the immediacy and personal relevance of preconception health knowledge, influencing their engagement with the topic, as they were not personally interested in it at the time of the research. Hossani et al., meanwhile, reported a much higher level of knowledge regarding the importance of folic acid in pregnant mothers in the UAE [18].

Most participants had never been pregnant (91.7%), which could have been attributable to the heavy load of their medical studies. Interestingly, many respondents did not plan to become pregnant (23.4%), which could be attributable to changing attitudes among Emirati women or other factors that require further research [21]. In this study, we found a high prevalence of genetic diseases (22%), which might be attributable to the high percentage of consanguineous marriages among Emirati citizens [22].

Regarding health awareness, the data also revealed that most female participants had never visited a gynecologist (58.0%) or had a Pap smear (78.0%), indicating a large gap in awareness of preventative healthcare measures. However, the reason for this could be related to guidelines in the UAE, where unmarried females cannot be examined bimanually, or a Pap smear is performed. While a notable number of participants actively engaged in physical activities (72.4%), there was still an overall lack of routine medical checkups. Such high levels of physical activity are promising, especially in countries where outdoor activity is severely limited by hot climates. The present study involved single individuals with limited reproductive experience and revealed significant gaps in their health awareness. A study conducted in Minya, Egypt, comprising 856 individuals, including 106 nurses, found that nurses had significantly higher knowledge and awareness regarding preconception health, which was attributed to their training in antenatal care [23]. While only 6.1% of the participants had experienced planned pregnancies, nurses showed more proactive attitudes toward pregnancy planning. This highlights a clear difference in knowledge of preconception care between nurses and medical students, emphasizing the need for targeted education to improve health awareness and preventive care practices.

In the present study, public understanding of how various lifestyle choices affect fertility and pregnancy was notably diverse. The overall level of knowledge about preconception health among medical students was low, which might have resulted from a lack of certain topics in the curriculum. The students attended only one lecture on preconception health in their fifth year of study. Moreover, the topic is not popular in the media and has not yet been addressed in secondary schools. Many people recognize the adverse effects of smoking and alcohol consumption during pregnancy as well as the importance of vitamin D. There is also widespread awareness of the benefits of infant formulas and the need for preconception preparation. However, there is a lack of knowledge regarding the importance of vaccinations before pregnancy, and many do not understand the critical role of folic acid in preconception care. Similarly, an article from Saudi Arabia highlighted the general understanding of how lifestyle choices such as smoking, alcohol consumption, diet, and weight can affect fertility and pregnancy [24]. Researchers have also noted the importance of prenatal vitamins (e.g., folic acid). There is also a common understanding of the need to adjust medications and diet during pregnancy to avoid harmful substances that might lead to malformations. The present study provides more in-depth knowledge, especially regarding awareness of the male fertility factor, which was not covered in the Saudi study [24]. An editorial article prepared by Qin and Xie highlights the importance of appropriate nutrition and supplement intake during pregnancy [25]. Both of those studies highlighted the importance of specific nutrients, such as vitamin D and folic acid, in preconception health. Our study covered a broader range of topics, such as vaccinations and male fertility, while the aforementioned article focused more on antenatal mothers. The differences in the results could be attributable to the target group (our study covered the reproductive age group aged 18–45, not just antenatal mothers) [25]. These findings contrast with those of Al-Hossani et al., who found that only 27% of pregnant women in the UAE had knowledge of preconception care, highlighting the potentially higher awareness among medical students [18]. A study conducted in Minya, Egypt, on the knowledge and attitudes of women and nurses regarding preconception care revealed a strong foundation for preconception awareness; however, 72.6% of nurses received training in antenatal care, and 60.4% provided preconception care [23]. In that study, nurses exhibited significantly higher knowledge and awareness of preconception health than women (non-nurses). However, both groups displayed hesitant attitudes toward pregnancy planning, highlighting the need for improved education regarding preconception health. Women’s knowledge was positively influenced by prior counseling and antenatal care, highlighting factors that could enhance preconception awareness.

In our study, the most notable finding from the WHOQOL questionnaire was that participants had a high QoL in all domains, with the highest ratings for environmental factors, suggesting that healthy food, low air pollution, safety, and stable living conditions significantly enhanced QoL. However, social relationships were rated the lowest, indicating challenges with interpersonal connections and social support. These results align with those of Chang et al., who found that environmental stability plays a crucial role in improving QoL [26]. Similarly, Singh et al. [27] found that in lower-income areas, strong social networks can compensate for weaker environmental conditions, emphasizing the influence of cultural and regional factors [27]. Such findings suggest that while environmental factors are essential for improving QoL, social connections are equally crucial, depending on the context. Addressing weaker areas through targeted interventions could create a more balanced improvement in QoL, especially in domains such as social relationships.

Our data revealed that many respondents relied on the Internet for information, and 50.7% used mobile applications for that purpose. A study of the Qatari population showed heavy reliance on the Internet, with 71.1% of the participants using the Internet as their primary source of health information [28]. Most participants were aged 18–34 (67.9%), while 30.1% were aged 35–59. There were notable gender differences in the use of the Internet as a source of health information, where 78.7% of those who used the Internet were female, and only 60.8% were male, which is in line with the demographics of the present study [28]. Similarly, our study demonstrated a heavy reliance on the Internet as a source of health-related information owing to its convenience and ease of use. Moreover, students prefer one or two sources of knowledge; therefore, designers of target information sources should be encouraged to provide comprehensive knowledge from a single source. These findings can direct our approaches to delivering health information related to preconception health using Internet sources, paying particular attention to mobile applications. Additionally, our study highlights the importance of accessible and familiar sources, such as the Internet and family, in shaping participants’ understanding. Traditional sources such as books and scientific journals did not have the same influence, which could indicate a shift toward more immediate and readily available information platforms. This suggests that for health education efforts to be effective, there could be value in utilizing commonly accessed sources, such as family discussions and online resources, to improve knowledge retention and impact. The analysis also derived interesting insights into the relationship between BMI and QoL. No significant relationship was observed between BMI and QoL across various domains, suggesting that BMI might not play a substantial role in shaping perceptions of well-being in this group. This lack of an association implies that factors other than BMI, such as lifestyle and mental health, might have stronger effects on how individuals perceive their QoL. It is worth noting, however, that in the present study, the median BMI was normal, which could have influenced the results.

Additionally, we found a positive correlation between knowledge level and QoL across all four domains. These findings suggest that using appropriate medical knowledge to influence healthy habits could increase global well-being.

Preconception health is critical for improving maternal and child health. Despite its importance, there are significant gaps in knowledge and awareness among individuals of reproductive age. Addressing these gaps through education and awareness is essential for achieving better health outcomes. This study lays the groundwork for understanding preconception health knowledge and attitudes among UAE medical students. Further analysis is needed to draw more comprehensive conclusions and to further compare the findings with those of other studies.

### 4.1. Limitations

This study has some limitations that might have influenced the results. First, our study population comprised medical students from a single university in the UAE. While this study provides valuable insights into the UAE context, its findings might not be fully generalizable to other countries because of variations in healthcare systems, cultural practices, and socioeconomic conditions. Moreover, the findings are specific only to medical students in one academic center that mostly includes students of Emirati origin. Another limitation is that the small group of participants represented only about 34% of medical students in the College of Medicine and Health Sciences. Moreover, online questionnaires comprising closed questions can produce bias, such as suggesting answers or influencing a particular health approach or inappropriate understanding of the question that happened with the question on the planned character of previous pregnancies.

### 4.2. Strengths

To our knowledge, this is the first study to examine levels of preconception knowledge among medical students in the Middle East and North Africa region. While the large percentage of Emirati nationals in this study offers culturally specific insights, it limits generalizability to other populations. Moreover, to create a future tool for reaching a larger group of recipients, we examined preferred sources of information. An additional benefit is the presentation of the influence of medical knowledge and attitudes on QoL.

## 5. Conclusions

Research suggests that lifestyle, health attitudes, and medical knowledge significantly influence individuals’ perceptions of their quality of life (QoL). Moreover, it highlights the importance of medical knowledge, not only for medical well-being but also for general well-being in all domains. Education can empower individuals to make informed health choices before conception, leading to healthier pregnancies and better outcomes for mothers and their children. The above emphasizes the need to develop a dedicated program (including an Internet platform) targeted at educating women on preconception care, particularly in the UAE in the early years of medical school. Once implemented to its full potential, couples’ education on preconception care can play a vital role in preventing poor neonatal health outcomes and undesirable adverse maternal health effects. Fostering strong social networks and support systems could be beneficial for preconceptual awareness and knowledge among medical students. This could involve using community-based programs and peer support groups to provide reliable information and emotional support, thereby enhancing both social connectedness and awareness. Encouraging discussions about preconception health in family or community settings could also help bridge gaps in social support and potentially improve overall preconception knowledge and preparedness.

## Figures and Tables

**Table 1:** Sociodemographic characteristics of the study group (*n* = 214).

Variable	Option	Count (*n*)	Percentage (%)
Gender	Woman	205	95.8
	Man	9	4.2
Age	<20 years	108	50.5
	20–25 years	103	48.0
	25–34 years	1	0.5
	35–44 years	1	0.5
	55–64 years	1	0.5
Nationality	Afghan	1	0.5
	Egypt	6	2.8
	Emirates	195	91.1
	Indian	1	0.5
	Lebanon	1	0.5
	Oman	1	0.5
	Pakistani	1	0.5
	Saudi	1	0.5
	Sudanese	2	0.9
	Syrian	1	0.5
	Tunisian	1	0.5
	Yemen	2	0.9
	Errors	2	1
Emirates	Abu Dhabi	159	74.3
	Ajman	4	1.9
	Fujairah	12	5.6
	Dubai	7	3.3
	Sharjah	2	0.9
	Ras Al Khaimah	15	7.0
	No data	2	1.4
Highest level of education	Secondary school	71	33.2
	Bachelor Degree	80	37.4
	Master Degree	1	0.5
	Diploma	57	26.6
	Errors	5	2.3
Household income per year	<50,000AED	71	33.2
	50,000–100,000 AED	47	22.0
	100,000–200,000 AED	27	12.6
	200,000–300,000 AED	15	7.0
	300,000–500,000 AED	20	9.3
	500,000–750,000 AED	11	5.1
	750,000–1,000,000 AED	13	6.0
	>1,000,000 AED	10	4.7
Marital status	Single	196	91.6
	Married	15	7.0
	Separated	1	0.5
	Divorced	2	0.9

**Table 2:** Medical characteristics and health awareness (*n* = 214).

Variable	Option	Count (*n*)	Percentage (%)
History of pregnancy	No	188	91.7
	Yes	7	3.4
	No data	10	4.9
	Total	205	100.0
	Not applicable (males)	9	
Number of children	0	59	27.6
	2	4	1.9
	8	1	0.5
	No data	150	70.1
	Total	214	100.0
Plan for future pregnancies	I have plans at present	61	28.5
	I am considering that in the next 1–2 years	24	11.2
	I am considering that in the next 3–5 years	27	12.6
	I don’t plan to have kids	50	23.4
	No data	52	24.3
	Total	214	100.0
Genetic diseases in the family	No	167	78.0
	Yes	47	22.0
	Total	214	100.0
Any current medical conditions	No	194	90.2
	Yes	20	9.3
	Total	214	100.0
Sport activity	No	59	27.6
	Yes	155	72.4
	Total	214	100.0
Frequency of sport activity	Every day	34	15.9
	A few times a week	71	33.2
	Once a week	26	12.1
	A few times a month	26	12.1
	Once a month	13	6,0
	A few times per year	18	8.4
	No data	26	12.1
	Total	214	100.0
Frequency of OBGYN visits	At least once a year	55	26.8
	At least once every 3 years	26	12.7
	At least once in 5 years	2	1.0
	I haven’t been to a gynecologist	119	58.0
	Errors	3	1.5
	Total	205	100.0
	Not applicable (males)	9	
Frequency of Pap Smear	At least once a year	28	13.7
	At least once every 3 years	14	6.8
	At least once in 5 years	3	1.5
	I have never done it before	160	78.0
	Total	205	100
	Not applicable	9	

**Table 3:** Level of knowledge (*n* = 214).

Question		Mark				
		−2	−1	0	1	2
Folic acid is important in preconception care	N	20	10	97	44	43
	%	9.3	4.7	45.3	20.6	20.1
Vitamin D is important in preconception care	N	28	4	35	66	81
	%	13.1	1.9	16.4	30.8	37.9
Smoking cigarettes may lower your fertility	N	18	11	59	73	53
	%	8.4	5.1	27.6	34.1	24.8
It is allowed to drink alcohol during pregnancy	N	4	12	27	13	158
	%	1.9	5.6	12.6	6	73.5
Obesity is not important in the preconception period	N	6	27	84	49	48
	%	2.8	12.6	39.3	22.9	22.4
You don’t have to be fully vaccinated before pregnancy	N	12	32	91	42	37
	%	5.6	15	42.5	19.6	17.3
Sometimes you have to change your medicaments before pregnancy	N	12	4	67	85	46
	%	5.6	1.9	31.3	39.7	21.5
Eating raw meat is safe during pregnancy	N	11	19	77	36	71
	%	5.1	8.9	36	16.8	33.2
Baby milk is as good as a mother’s natural milk	N	29	35	51	59	40
	%	13.6	16.4	23.8	27.6	18.7
You don’t have to prepare for pregnancy if you are young and healthy	N	9	31	63	73	38
	%	4.2	14.5	29.4	34.1	17.8
Some Genetic diseases need to be diagnosed before conception	N	12	4	59	82	57
	%	5.6	1.9	27.6	38.3	26.6
An unhealthy lifestyle may lower male fertility	N	14	5	70	67	58
	%	6.5	2.3	32.7	31.3	27.1

**Table 4:** Overall level of knowledge (*n* = 214).

Level of Knowledge	*n*	%
1 (lack of knowledge	47	22.0
2 (low level of knowledge)	104	48.6
3 (good level of knowledge)	63	29.4
Total	214	100.0

**Table 5:** Quality of life of the participants (WHOQOL results) (n = 214).

	N	Minimum	Maksimum	Med	SD
Physical_domain	214	48	140	93.40	18.14
Psychological_domain	214	40	120	78.13	16.67
Social_relationship_domain	214	12	60	40.45	10.33
Enwironment_domain	214	32	160	112.30	27.18

**Table 6:** Correlation between level of knowledge and quality of life (*n* = 214).

		Physical Domain	Psychological Domain	Social Relationship Domain	Environment Domain
Overall level of knowledge	r	0.263 **	0.230 **	0.224 **	0.419 **
	*p*	0.0001	0.001	0.001	0.0001
	N	214	214	214	214

** Correlation is significant at the level of 0.01 (bilateral).

## Data Availability

The original contributions of this study are included in the article; further inquiries can be directed to the corresponding author.
